# MET gene alterations predict poor survival following chemotherapy in patients with advanced cancer

**DOI:** 10.3389/pore.2022.1610697

**Published:** 2022-11-22

**Authors:** Jihoon Ko, Jaeyun Jung, Seung Tae Kim, Jung Yong Hong, Sehhoon Park, Joon Oh Park, Young Suk Park, Ho Yeong Lim, Soomin Ahn, Kyoung-Mee Kim, Won Ki Kang, Jeeyun Lee

**Affiliations:** ^1^ Division of Hematology-Oncology, Department of Medicine, Samsung Medical Center, School of Medicine, Sungkyunkwan University, Seoul, South Korea; ^2^ Department of Pathology and Translational Genomics, Samsung Medical Center, School of Medicine, Sungkyunkwan University, Seoul, South Korea

**Keywords:** next-generation sequencing, oncogene, overall survival analysis, MET, MET alterations, chemotherapy, cancer

## Abstract

**Background:** To aid in oncology drug development, we investigated MET proto-oncogene receptor tyrosine kinase gene aberrations in 2,239 oncology patients who underwent next-generation sequencing (NGS) in clinical practice.

**Materials and methods:** From November 2019 to January 2021, 2,239 patientswith advanced solid tumors who visited oncology clinics underwent NGS. The NGS panel included >500 comprehensive NGS tests using archival tissue specimens. Programmed death-ligand 1(PD-L1) 22C3 assay results and clinical records regarding initial chemotherapy were available for 1,137 (50.8%) and 1,761 (78.7%) patients, respectively for overall survival (OS) analysis.

**Results:** The 2,239 patients represented 37 types of cancer. The NGS panel included >500 genes, microsatellite instability status, tumor mutational burden, and fusions. The most common cancer types were colorectal (*N* = 702), gastric (*N* = 481), and sarcoma (*N* = 180). MET aberrations were detected in 212 patients. All MET-amplified tumors had microsatellite stable status, and 8 had a high tumor mutational burden. Of 46 patients with MET-amplified cancers, 8 had MET-positive protein expression by immunohistochemistry (2+ and 3+). MET fusion was detected in 10 patients. Partner genes of MET fusion included ST7, TFEC, LRRD1, CFTR, CAV1, PCM1, HLA-DRB1, and CAPZA2. In survival analysis, patients with amplification of MET gene fusion had shorter OS and progression-free survival (PFS) than those without. Thus, MET aberration was determined to be a factor of response to chemotherapy.

**Conclusion:** Approximately 2.1% and 0.4% of patients with advanced solid tumors demonstrated MET gene amplification and fusion, respectively, and displayed a worse response to chemotherapy and significantly shorter OS and PFS than those without MET gene amplification or fusion.

## Introduction

MET proto-oncogene receptor tyrosine kinase plays a pivotal role in multiple cellular processes such as carcinogenesis and tumor progression in several solid tumor types [1,2,3]. Studies have shown that dysregulation of MET signaling pathway including gene amplification, overexpression of the ligand and/or receptor, autocrine signaling, and paracrine signaling has been indicated as a cancer-associated mechanism [4,5,6,7]. Because MET plays a critical role in cancer progression, its inhibition could have a substantial impact on the treatment outcome of patients with solid tumors with an aberrant MET pathway. For instance, approximately 5% of patients with gastric cancer (GC) have increased copy numbers (no. of gene copies >4) of the MET gene [8,9,10,11,12]. Moreover, the MET Gastric trial, in which patients with GC with MET overexpression (immunohistochemistry [IHC], 2+/3+) received onartuzumab, an anti-MET monoclonal antibody, plus FOLFOX did not demonstrate significantly improved survival [13]. Additionally, the VIKTORY basket trial observed a promising overall response rate of 50% among 20 patients with MET-amplified GC [14] using savolitinib, a MET tyrosine kinase inhibitor (TKI). Several trials of MET-targeted agents are ongoing, specifically for patients with GC [10, 11].

MET amplification has been reported in 5%–26% of cases to be implicated in the acquired resistance to epithelial growth factor receptor TKIs [15]. Moreover, MET alterations have been identified in primary tumors and metastatic lesions of several types of cancer, including head and neck, papillary renal cell carcinoma, liver, ovarian, and non-small cell lung cancer [16]. Elevated levels of the HGF receptor ligand or overexpression of MET is often associated with resistance to chemotherapy and radiotherapy [17]. Overall, MET dysregulation is recognized as a negative prognostic factor in many solid cancers. However, few studies have provided real-world data of MET dysregulation across many types of cancer. Additionally, more countries including the United States, Korea, and Japan, are adopting the use of next generation sequencing (NGS) in clinical practice for patients with metastatic cancer [14, 18, 19]. Therefore, understanding the prevalence of MET-aberrant solid tumor types is vital to optimize clinical trial design.

This study aimed to investigate the incidence of MET aberrations, including copy number variations (CNVs), especially gene amplification, and/or fusions using a 500-gene NGS panel as real-world, pan-cancer data. Furthermore, we analyzed the impact of MET alterations on response to chemotherapy and survival.

## Materials and methods

### Ethical approval

This retrospective study was approved by the Institutional Review Board of Samsung Medical Center (IRB File No. 2021-09-052). All patients who participated in this study provided written informed consent prior to NGS, and additional informed consent was waived by the IRB. This study was performed in accordance with the principles of the Declaration of Helsinki and Korean Good Clinical Practice guidelines.

### DNA extraction

Tumor regions were micro-dissected for most tumor tissues, except for the samples used in genomic DNA extraction. Genomic DNA was isolated from formalin-fixed paraffin-embedded (FFPE) tissue fragments and purified using AllPrep DNA/RNA FFPE Kit (Qiagen, Venlo, Netherlands). DNA concentration was measured using a Qubit dsDNA HS assay kit (Thermo Fisher Scientific, Waltham, MA, United States), and 40 ng DNA was used as the input for library preparation. The DNA integrity number, a measure of DNA fragment size and quality, was determined using the Genomic DNA ScreenTape assay on an Agilent 2,200 TapeStation system (Agilent Technologies, Santa Clara, CA, United States).

### Library preparation and data analysis

The DNA library was prepared using a hybrid capture-based TruSight Oncology 500 (TSO 500) DNA/RNA NextSeq Kit according to the manufacturer’s protocol. During library preparation, enrichment chemistry was optimized to capture nucleic acid targets from FFPE tissues. Unique molecular identifiers were used in TSO 500 analysis to determine the unique coverage at each position and reduce any background noise caused by sequencing and deamination artifacts in the samples. This technique enables thxe detection of variants at low variant allele frequencies while simultaneously suppressing errors, thereby providing high specificity.

Sequence data were analyzed for clinically relevant classes of genomic alterations, including single nucleotide variants (SNVs), small insertions and deletions (indels), CNVs, and rearrangements/fusions. Results of SNVs and small indels with a variant allele frequency of <2% were excluded. Average CNVs of more than four were considered as gain and those less than one were considered as loss. Only gain (gene amplification) was analyzed in the TSO 500-CNV analysis, and RNA translocation-supporting reads of >4–12 were considered as translocation, which was dependent on the quality of the sample. Data outputs exported from the TSO 500 pipeline (Illumina, San Diego, CA, United States) were annotated using the Ensembl Variant Effect Predictor Annotation Engine with information from databases, such as dbSNP, gnomAD genome and exome, 1,000 genomes, ClinVar, COSMIC, RefSeq, and Ensembl [20]. The processed genomic changes were categorized according to a four-tier system proposed by the American Society of Clinical Oncology/College of American Pathologists and annotated with proper Ref. [21]. The TSO 500 pipeline (Illumina) was used to evaluate tumor mutational burden (TMB) and microsatellite instability (MSI) statuses [20]. TMB was calculated by excluding any variant with an observed allele count ≥10 in any of the GnomAD exome, genome, and 1,000 genomes databases, and including variants in the coding region (RefSeq Cds), variant frequency ≥5%, coverage ≥50×, SNVs, and indels, as well as including and excluding nonsynonymous and synonymous variants. The effective panel size for TMB was the total coding region with coverage >50×. MSI was calculated from the microsatellite sites to evaluate instability relative to a set of normal baseline samples based on entropy metrics. The percentage of unstable MSI sites out of the total assessed MSI sites was reported as a sample-level microsatellite score.

### Statistical analysis

Data are presented as the mean ± SD. All statistical analyses were performed using GraphPad Prism 8.0 (GraphPad Software, San Diego, CA, United States; http://www.graphpad.com/). Statistical significance was set at *p* < 0.05. All statistical tests were two-sided. Correlations between clinicopathologic features were analyzed using a *t*-test, Fisher exact test, or one-way analysis of variance, as appropriate. Overall survival (OS) was defined as the time from the first treatment to the date of death. Progression-free survival (PFS) was defined as the time from the first treatment to the date of disease progression or death from any cause. The Kaplan-Meier method was used to analyze all survival events, and the 95% confidence interval for the median time to each event was computed. All statistical analyses were performed using Prism 8 (GraphPad Software, San Diego, CA, United States; http://www.graphpad.com/) or R for windows (version 4.1.2, https://cran.r-project.org/bin/windows/base/). RStudio desktop 1.4 was used for drawing all graphics (RStudio Team, Boston, MA, United States; https://www.rstudio.com/products/rstudio/download/).

## Results

### Patient cohort

**FIGURE 1:**
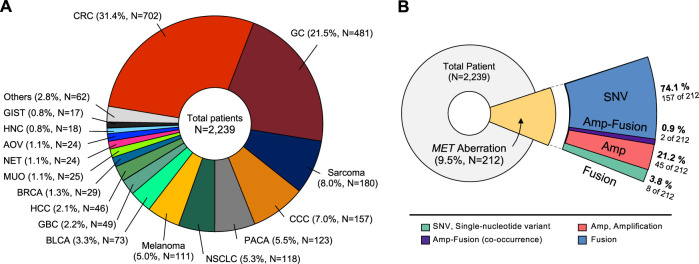
Classification of tumor types in patients enrolled in the study. **(A)** Distribution of tumor types in 2,239 patients. The most common type was colorectal cancer (31.4%), followed by gastric cancer (21.5%), and sarcoma (8.0%). **(B)** Diagram showing proportion of MET aberrations in the cohort, including amplification, fusion, and SNVs. CRC, Colorectal cancer; GC, Gastric cancer; CCC, cholangiocellular carcinoma; PACA, pancreatic cancer; NSCLC, non-small cell lung cancer; BLCA, bladder cancer; GBC, gallbladder cancer; HCC, hepatocellular carcinoma; BRCA, breast cancer; MUO, metastasis of unknown origin; NET, neuroendocrine tumor; AOV, ampulla of vater; HNC, head and neck cancer; GIST, gastrointestinal stromal tumor.

We conducted a study of aberrant genes utilizing NGS with a panel targeting more than 500 oncogenes (TSO500, Illumina) in a total of 2,239 patients with advanced cancer (Table 1). By gender, there were 1,342 males (60.0%) and 894 females (40.0%). And as for TMB, 332 cases (14.8%) of High-TMB (≥13 mut/Mb) and 1,905 cases (85.2%) of Low-TMB were found. In the case of MSI, 13 cases (0.6%) with low-MSI, 38 cases (1.7%) with high-MSI, and most of the rest were MSS (microsatellite stability). In this cohort, 38 types of cancer were identified (Table 2). The most common cancers were colorectal cancer (702 cases, 31.4%), gastric cancer (481 cases, 21.5%), and sarcoma (180 cases, 8.0%) in that order (Figure 1A). MET aberrations, known to be strongly correlated with tumorigenesis and metastasis, were found in a total of 212 patients. The incidence of alterations were: MET gene amplification (21.2%), MET fusion (3.8%), co-occurrence of amplification and fusion (0.9%), and SNV (74.1%) (Figure 1B).

**TABLE 1:** Clinicopathologic features of study patient.

Sex	
Male	1,342 (60.0%)
Female	894 (40.0%)
TMB	
Low	1,905 (85.2%)
High	332 (14.8%)
MSI	
Low	13 (0.6%)
High	38 (1.7%)
MSS	2,187 (97.7%)
CNV	2,515
Tier I/II	841 (33.4%)
Tier III	1,674 (66.6%)
Fusion	383
Tier I/II	149 (38.9%)
Tier III	234 (61.1%)
SNV	44,534
Tier I/II	1,663 (3.7%)
Tier III	42,871 (96.3%)

**TABLE 2:** Tumor types in the cohort.

Tumor type	#Patient
Adrenocortical carcinoma	1 (0.04%)
AOV cancer (Ampulla of Vater)	24 (1.07%)
Bladder cancer	73 (3.26%)
Brain tumor	1 (0.04%)
Breast cancer	29 (1.30%)
CCC (Cholangiocellular carcinoma)	157 (7.01%)
Cervical cancer	1 (0.04%)
Colorectal cancer	702 (31.35%)
Duodenal cancer	6 (0.27%)
EAC cancer (Esophageal adenocarcinoma)	1 (0.04%)
Esophageal cancer	1 (0.04%)
Fallopian tube cancer	1 (0.04%)
Gallbladder cancer	49 (2.19%)
Gastric cancer	481 (21.48%)
GIST (Gastrointestinal stromal tumor)	17 (0.76%)
HCC (Hepatocellular carcinoma)	46 (2.05%)
Head and Neck cancer	18 (0.80%)
Kidney cancer	9 (0.40%)
Malignant solitary fibrous tumor	5 (0.22%)
Malignant thymoma	1 (0.04%)
Melanoma	111 (4.96%)
Merkel cell carcinoma	1 (0.04%)
MUO (Metastasis of unknown origin)	25 (1.12%)
NET (Neuroendocrine tumors)	24 (1.07%)
Neurofibromatosis	1 (0.04%)
NSCLC (Non-small cell lung cancer)	119 (5.31%)
Ovarian cancer	9 (0.40%)
Paget’s disease	2 (0.09%)
Pancreatic cancer	123 (5.49%)
Prostate cancer	5 (0.22%)
Sarcoma	180 (8.04%)
SCLC (Small cell lung cancer)	1 (0.04%)
Skin cancer	4 (0.18%)
Small bowel cancer	4 (0.18%)
Thymic carcinoma	2 (0.09%)
Thyroid cancer	3 (0.13%)
Tracheobronchial adenoid cystic carcinoma	1 (0.04%)
Uterine cancer	1 (0.04%)
	**2,239**

### MET gene amplification

**FIGURE 2:**
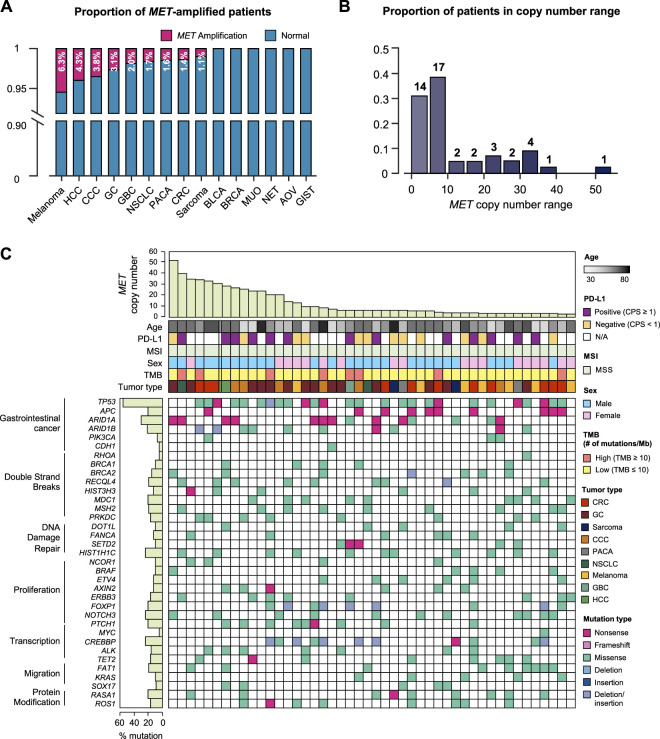
Genomic landscape of cancer patients with MET copy number variations. **(A)** Bar graph showing the incidence of MET amplification by tumor type. **(B)** The distribution of MET-amplified patients according to copy number. **(C)** The landscape of patients’ genomic profiles. Top panel, copy number of each patient; middle panel, clinical information including age, PD-L1 expression (IHC), MSI, sex, TMB, and cancer type; bottom panel, oncoprint showing SNV type of genes listed at the left. Left panel, the alterations rate of each gene in total samples (*N* = 46) with biological function annotation. IHC, immunohistochemistry; MSI, microsatellite instability; TMB, tumor mutational burden; SNV, single nucleotide variant.

We specifically investigated MET gene amplification in MET aberration involving copy number and structural changes as well as nucleotide variants. In this case, we collected the cases more than 4 copies of MET gene. The patients’ age ranged from 30 to 82 years with a median of 58 years, and no gender bias was observed. Of the 47 patients with MET amplification, 14 had GC (2.9% of total GC patients), 10 (1.4%) had colorectal cancer, 7 (6.3%) had melanoma, 6 (3.8%) had cholangiocellular carcinoma (CCC), 2 each had sarcoma (1.1%); hepatocellular carcinoma (HCC) (4.3%); pancreatic cancer (1.6%); and non-small cell lung cancer (1.7%), and gallbladder cancer (4.1%). In addition, we investigated tumors with a high incidence of MET amplification. The incidence of MET amplification among melanoma patients was 6.3%, which showed a high expression rate within the group. Furthermore, 4.3% of HCC patients, 3.8% of CCC patients, and 3.1% of GC patients showed a high incidence of MET amplification (Figure 2A). We analyzed the range of number of amplified MET copies in the patient group. MET copy numbers ranged from 3.1 to 52.2 with a median of 7.2. The highest level of MET copy number is 52.2 identified in a GC patient (Figure 2B). Moreover, all MET-amplified tumors had MSS status and 4.3% (*N* = 2) of cases had a high TMB status (≥13 mut/Mb) (TMB ranged from 0.8 to 15.7 with median of 7.0). In the pan-tumor analysis of patients with PD-L1 IHC data available, no correlation between PD-L1 expression and MET amplification was observed. The overall genomic landscape including known oncogenes in the MET-amplified patient cohort is provided in Figure 2C. Briefly, most MET-amplified patients had concurrent mutations in TP53, APC, and ARID1A across all cancer types surveyed in this study (Figure 2C).

### MET gene fusion

**FIGURE 3:**
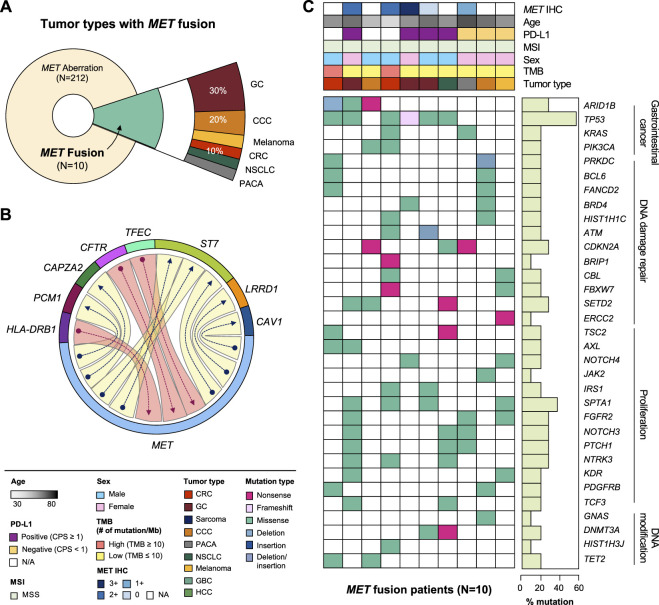
Clinicopathological landscape of patients with MET fusions. **(A)** Diagram showing the distribution of patients and tumor types with MET fusion. **(B)** Chord diagram exhibiting flows or connections between the MET gene and other partner genes. **(C)** Landscape of the patient’s genomic profile. Upper panel, clinical information regarding MET expression (IHC), age, PD-L1, MSI, sex, TMB, and tumor type; lower panel, SNV profile of genes indicated at the left panel; right panel, mutation rate of each gene and biological functional annotation. IHC, immunohistochemistry; MSI, microsatellite instability; TMB, tumor mutational burden; SNV, single nucleotide variant.

MET fusion occurs due to gene rearrangement and has been observed in various types of cancer. As mentioned above, approximately 0.4% (10 of 2,239) of the patients in this study had MET fusion including cooccurrence of amplification and fusion cases (Figure 3A). The partner genes of MET fusion included ST7, TFEC, LRRD1, CFTR, CAV1, PCM1, CAPZA2, and HAL-DRB1 (Figure 3B). The most frequent gene fusion occurred between MET and ST7. Excluding fusion with PCM1 and CAPZA2, all breaking began in the middle of the MET gene whose front part was fused to the back part of the partner gene. Regarding mutation type, missense MET alterations was dominant across all tumor types. Moreover, several cases of frameshift deletion, in-frame deletion, and nonsense mutations were observed. The most frequent MET SNVs were MET L211T (*N* = 24) and A1381T (*N* = 19). Finally, most patients had concurrent mutations in TP53, KRAS, and ARID1B (Figure 3C).

### MET Single-Nucleotide variations

**FIGURE 4:**
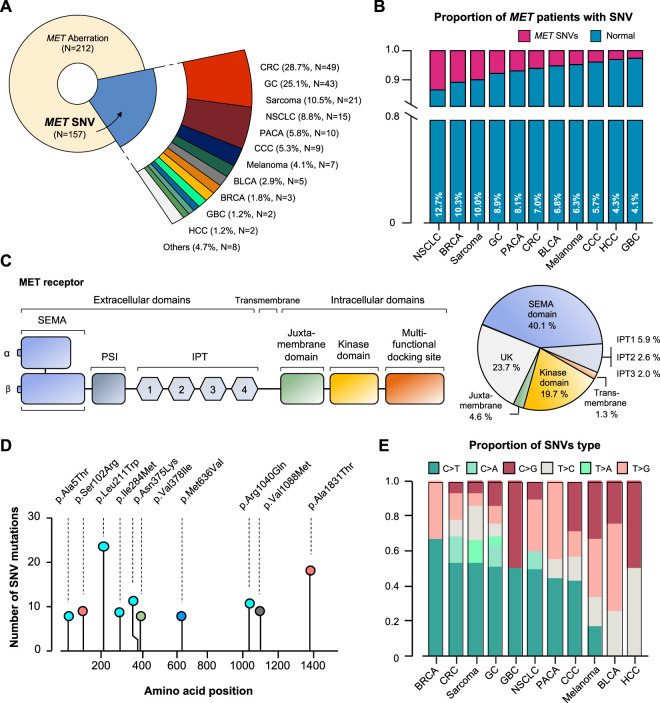
MET-gene-associated SNV profiles. **(A)** Tumor types and proportions of patients with SNV in the MET gene. **(B)** The percentage of SNV samples for each cancer (SNV in MET gene). **(C)** Structure of MET protein and identified MET-gene-associated SNV in the group. **(D)** The signature of nucleotide changes showing the ratio of transition to transversion. **(E)** Bar graph showing the SNV type ratio by tumor types. SNV, single nucleotide variant. UK, unknown.

Of the 157 MET SNV patients, 28.7% had CRC (*N* = 49), 25.1% had GC (*N* = 43), 10.5% had sarcoma (*N* = 21), 8.8% had NSCLC (*N* = 15), and other tumors (Figure 4A). It was analyzed that the incidence of cancer caused by MET SNV was high in the order of NSCLC (12.7%), breast cancer (BRCA; 10.3%), sarcoma (10.0%), and GC (8.9%) (Figure 4B). We performed single nucleotide variant analysis of MET protein. Of 157, 40.1% (*n* = 61) were detected mainly in the semaphorin (Sema) domain (E168D) (*N* = 56 for exon 2; *n* = 5 for exon 1). And, MET exon 14 juxtamembrane splicing mutations (*N* = 7) were detected (GC 3; Sarcoma 2; CRC 1; and CCC 1). SNV alterations were identified in the MET IPT/TIG (Ig-like, plexins, transcription factors) domain (exon 5-9). In addition, mutations were detected in the intracellular tyrosine kinase domain of MET (exon 16,18-21) in 19.7% (Figure 4C). We conducted an investigation into sequence variations of amino acids. The p. L211W variant located in coding exon 1 of the MET gene (also known as c.632T>G) was found to have the most T to G substitution at nucleotide position 632 (Figure 4D). We plotted incidence by SNV type relative to tumor type (Figure 4E).

### Patients with MET gene aberration showed poor survival

**FIGURE 5:**
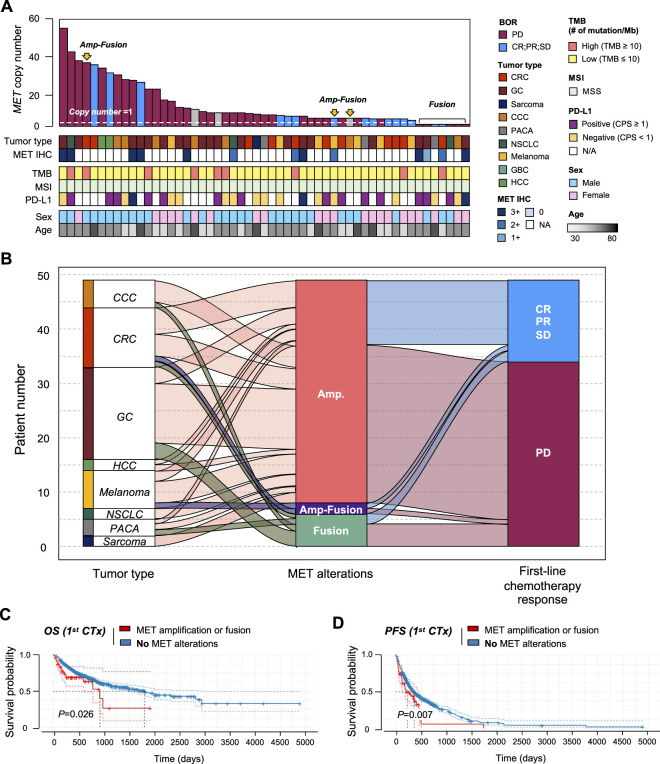
Correlation between MET gene alterations and response to first-line chemotherapy. **(A)** Cancer genomic landscape refers to the distribution pattern of MET gene alterations across the genome in tumor types. **(B)** Alluvial diagram showing the distribution of MET aberration in tumor types and the best response to first-line chemotherapy. Kaplan-Meier curves showing **(C)** overall survival (OS) and **(D)** progression-free survival (PFS) with (amplification of fusion) or without MET gene alterations.

We established the genomic landscape of patients with MET aberrations (Figure 5A). In an alluvial diagram, patients with MET amplification or fusion showed a correlation with disease progression after first-line chemotherapy (29/41 patients with MET amplification had progressive disease (PD); 4/6 patients with MET fusion had PD) (Figure 5B). Moreover, in the survival curve, patients with MET CNV amplification or fusion showed significantly shorter OS (*p* = 0.026) and PFS (*p* = 0.007). Finally, we compared patients with CNV amplification in the MET gene to those with amplification in any gene. In this comparison, MET-CNV patients showed significantly shorter OS (*p* = 0.0039) and PFS (*p* = 0.016) than patients with amplification in any gene, suggesting that MET amplification is an influential factor for survival following first-line chemotherapy (Figure 5C).

## Discussion

In this study, we comprehensively investigated the incidence of MET alterations (gene amplification, SNV, and fusion) in a consecutive series of 2,239 patients with cancer who were candidates for palliative chemotherapy. Our cohort represented 37 different types of cancer. We found the incidence of MET amplification to be 2.1% (*N* = 46) and that of MET fusion to be 0.4% (*N* = 10), while 0.1% of patients (*N* = 3) had both MET amplification and fusion, simultaneously. Additionally, MET amplification was found in nine types of cancer. The range of copy numbers varied from 3.1 to 52.2. While most samples demonstrated <10, many samples displayed ≥10 copy numbers. Additionally, we observed a correlation between high copy number and probability of progressive disease. Furthermore, patients with MET aberrations demonstrated a shorter OS and PFS compared to those without. Finally, because patients with MET amplification showed a worse response to chemotherapy and no improved response to immuno-oncology therapy, a new targeted therapy was warranted for such patients.

Until recently, MET fusion, such as KIF5B-MET, MET-ATXN7L1, and HLA-DRB1-MET had mainly been reported in lung cancer. In our study, MET was most commonly fused with ST7 followed by CFTR, PCM1, LRRD1, CAV1, CAPZA2, and TEFC. Whether patients with MET fusion (according to NGS) or MET-negativity (according to IHC) respond to MET inhibitors warrants further investigation in future trials [22].

MET-amplified cases (*N* = 11) were confirmed by IHC, and approximately one-third of the patients with MET fusion had MET overexpression at the protein level (Supplementary Figure S1). We employed one of the most commonly used NGS panels in the clinic, namely, TSO 500 (Illumina). Although MET IHC and NGS results showed a strong correlation, more clinics are adopting NGS as a screening platform for oncology patients.

## Conclusion

We identified MET gene aberrations in a cohort of Korean patients at the Samsung Medical Center Precision Oncology Clinic. NGS screening identified 46 patients (2.1%) with MET amplification, 10 (0.4%) with MET fusion, and 3 (0.1%) with concurrent amplification and fusion. Aberrant MET genes appeared in various cancers in the following order: gastric cancer (N = 17, 32.1%), colorectal cancer (*N* = 11, 20.8%), and CCC (*N* = 7, 13.2%). MET amplification and fusion occurred concurrently in patients with colorectal cancer and CCC. Additionally, patients with MET aberration (amplification or fusion) demonstrated significantly shorter OS and PFS. Based on our data, NGS panels, particularly TSO 500, may be a feasible, real-world practice for identifying MET aberrations in the clinic. Our data can be used as a guideline for the design of future clinical trials of MET inhibitors.

## Data Availability

The data generated in this study are available within the article and its Supplementary Material.

## References

[1] Maulik G Shrikhande A Kijima T Ma-P C Morrison P Salgia R. Role of the hepatocyte growth factor receptor, c-Met, in oncogenesis and potential for therapeutic inhibition. Cytokine Growth Factor Rev (2002) 13(1):41–59. 10.1016/S1359-6101(01)00029-6 11750879

[2] Lee J Seo JW Jun HJ Ki CS Park SH Park YS Impact of met amplification on gastric cancer: possible roles as a novel prognostic marker and a potential therapeutic target. Oncol Rep (2011) 25(6):1517–24. 10.3892/or.2011.1219 21424128

[3] Lee SJ Lee J Park SH Park JO Lim HY Kang WK c-MET overexpression in colorectal cancer: a poor prognostic factor for survival. Clin Colorectal Cancer (2018) 17(3):165–9. 10.1016/j.clcc.2018.02.013 29576428

[4] Janjigian YY Tang LH Coit DG Kelsen DP Francone TD Weiser MR MET expression and amplification in patients with localized gastric cancer. Cancer Epidemiol Biomarkers Prev (2011) 20(5):1021–7. 10.1158/1055-9965.EPI-10-1080 21393565 PMC3690490

[5] An X Wang F Shao Q Wang F Wang Z Chen C MET amplification is not rare and predicts unfavorable clinical outcomes in patients with recurrent/metastatic gastric cancer after chemotherapy. Cancer (2014) 120(5):675–82. 10.1002/cncr.28454 24804300

[6] Peruzzi B Bottaro DP. Targeting the c-Met signaling pathway in cancer. Clin Cancer Res (2006) 1212:3657–60. 10.1158/1078-0432.CCR-06-0818 16778093

[7] Moosavi F Giovannetti E Peters GJ Firuzi O. Combination of HGF/MET-targeting agents and other therapeutic strategies in cancer. Crit Rev Oncol Hematol (2021) 160:103234. 10.1016/j.critrevonc.2021.103234 33497758

[8] Lee H Kim M Lee H Jung E Yang H Lee B MET in gastric carcinomas: comparison between protein expression and gene copy number and impact on clinical outcome. Br J Cancer (2012) 107(2):325–33. 10.1038/bjc.2012.237 22644302 PMC3394975

[9] Inokuchi M Otsuki S Fujimori Y Sato Y Nakagawa M Kojima K. Clinical significance of MET in gastric cancer. World J Gastrointest Oncol (2015) 7(11):317–27. 10.4251/wjgo.v7.i11.317 26600931 PMC4644854

[10] Kawakami H Okamoto I Arao T Okamoto W Matsumoto K Taniguchi H MET amplification as a potential therapeutic target in gastric cancer. Oncotarget (2013) 4(1):9–17. 10.18632/oncotarget.718 23327903 PMC3702203

[11] Chen C-T Kim H Liska D Gao S Christensen J Weiser M. MET activation mediates resistance to lapatinib inhibition of HER2-amplified gastric cancer cells. Mol Cancer Ther (2012) 11(3):660–9. 10.1158/1535-7163.MCT-11-0754 22238368 PMC4209288

[12] Ha SY Lee J Kang SY Do IG Ahn S Park JO MET overexpression assessed by new interpretation method predicts gene amplification and poor survival in advanced gastric carcinomas. Mod Pathol (2013) 26(12):1632–41. 10.1038/modpathol.2013.108 23807774

[13] Shah MA Cho J-Y Tan IB Tebbutt NC Yen CJ Kang A A randomized phase II study of folfox with or without the MET inhibitor onartuzumab in advanced adenocarcinoma of the stomach and gastroesophageal junction. Oncologist (2016) 21(9):1085–90. 10.1634/theoncologist.2016-0038 27401892 PMC5016069

[14] Lee J Kim ST Kim K Lee H Kozarewa I Mortimer PGS Tumor genomic profiling guides patients with metastatic gastric cancer to targeted treatment: the VIKTORY umbrella trial. Cancer Discov (2019) 9(10):1388–405. 10.1158/2159-8290.CD-19-0442 31315834

[15] Bang YJ Su WC Schuler M Nam DH Lim WT Bauer TM Phase 1 study of capmatinib in MET‐positive solid tumor patients: dose escalation and expansion of selected cohorts. Cancer Sci (2020) 111(2):536–47. 10.1111/cas.14254 31778267 PMC7004521

[16] Lorenzato A Olivero M Patanè S Rosso E Oliaro A Comoglio PM Novel somatic mutations of the MET oncogene in human carcinoma metastases activating cell motility and invasion. Cancer Res (2002) 62(23):7025–30. 12460923

[17] Raghav KPS Gonzalez-Angulo AM Blumenschein GR Jr. Role of HGF/MET axis in resistance of lung cancer to contemporary management. Transl Lung Cancer Res (2012) 1(3):179–93. 10.3978/j.issn.2218-6751.2012.09.04 25806180 PMC4367559

[18] Mukai Y Ueno H. Establishment and implementation of cancer genomic medicine in japan. Cancer Sci (2021) 112(3):970–7. 10.1111/cas.14754 33289217 PMC7935799

[19] Fujii S Magliocco AM Kim J Okamoto W Kim JE Sawada K International harmonization of provisional diagnostic criteria for ERBB2-amplified metastatic colorectal cancer allowing for screening by next-generation sequencing panel. JCO Precis Oncol (2020) 4:6–19. 10.1200/PO.19.00154 35050726

[20] Pestinger V Smith M Sillo T Findlay JM Laes JF Martin G Use of an integrated pan-cancer oncology enrichment next-generation sequencing assay to measure tumour mutational burden and detect clinically actionable variants. Mol Diagn Ther (2020) 24(3):339–49. 10.1007/s40291-020-00462-x 32306292 PMC7264086

[21] Karasaki T Nakajima J Kakimi K. Neoantigens and whole-exome sequencing. Gan To Kagaku Ryoho (2016) 43(7):791–7. 27431622

[22] Comoglio PM Giordano S Trusolino L. Drug development of MET inhibitors: targeting oncogene addiction and expedience. Nat Rev Drug Discov (2008) 7(6):504–16. 10.1038/nrd2530 18511928

