# Supplementary material for: MET gene alterations predict poor survival following chemotherapy in patients with advanced cancer
Source: Pathol Oncol Res. 2022 Nov 22;28:1610697. doi: 10.3389/pore.2022.1610697 (PMC9722768; doi:10.3389/pore.2022.1610697)

Supplementary Figure

Supplementary Figure 1. Immunohistochemical images of representative MET overexpression cases.

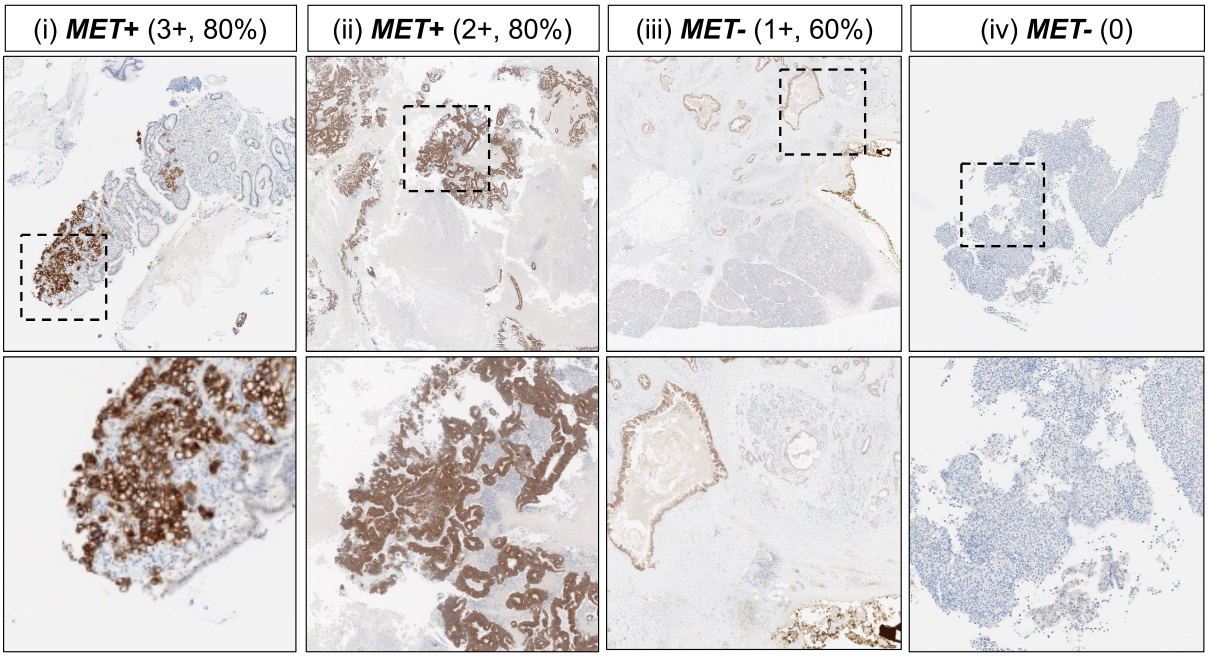

Supplement: Supplementary file 1 [file Image1.pdf]
